# Capsule and PspA Cooperatively Confer Resistance of *Streptococcus pneumoniae* to the Human Defensin HNP-1

**DOI:** 10.3390/ijms27072975

**Published:** 2026-03-25

**Authors:** Maria Eduarda Pereira Mendes, Thalita Bastos de Freitas e Silva, Rebeca Faria, Kelvin Gattinoni, Bruna Terribile, Giulia Destro, Lucio F. C. Ferraz, Anders P. Hakansson, Carlos J. Orihuela, Juliana Mozer Sciani, Thiago R. Converso, Michelle Darrieux

**Affiliations:** 1 Laboratório de Microbiologia Molecular e Clínica, Universidade São Francisco, Bragança Paulista 12916-900, SP, Brazil; duda.mendes017@gmail.com (M.E.P.M.); giuliavdestro@gmail.com (G.D.); lucio.ferraz@usf.edu.br (L.F.C.F.);; 2 Division of Experimental Infection Medicine, Department of Translational Medicine, Lund University, 221 84 Lund, Sweden; anders_p.hakansson@med.lu.se; 3 Department of Microbiology, Heersink School of Medicine, The University of Alabama at Birmingham, Birmingham, AL 35294, USA; corihuel@uab.edu; 4 Laboratório de Produtos Naturais, Universidade São Francisco, Bragança Paulista 12916-900, SP, Brazil

**Keywords:** pneumococcus, AMPs, HNP, defensin, PspA, capsule

## Abstract

*Streptococcus pneumoniae* resists host defenses through multiple surface factors, yet their specific contribution to protection against antimicrobial peptides remains incompletely understood. We examined the role of pneumococcal surface protein A (PspA) and the polysaccharide capsule in protection against the human defensin HNP-1. PspA conferred increased resistance to HNP-1-induced killing, shown by a decreased killing in the presence of purified recombinant PspA and an increased sensitivity when PspA was deficient from the surface of strains of two different genetic backgrounds or when anti-PspA antibody was present. The capsule also conferred protection against HNP-1, which was serotype-dependent, with type 2 protecting better than type 4, and free polysaccharides acted as decoys by sequestering HNP-1. Removal of surface PspA from capsule-deficient mutants revealed additive contributions of both factors to survival. Molecular docking analysis suggests a potential electrostatic interaction between PspA and HNP-1. These findings highlight the independent and complementary roles of PspA and the capsule in pneumococcal resistance to HNP-1 and provide novel insights that may inform future vaccine design and antimicrobial strategies.

## 1. Introduction

*Streptococcus pneumoniae* remains a major global pathogen responsible for localized and systemic infections, such as otitis media, sinusitis, pneumonia, and meningitis [1]. Its pathogenic success is largely due to its ability to asymptomatically colonize the nasopharyngeal mucosa, from where it can disseminate and cause disease [2]. An important factor contributing to pneumococcal dispersion and spread from the upper respiratory tract is coinfection with viruses, particularly influenza, which damages the mucosal barrier and triggers genetic changes in the bacteria, resulting in a more invasive phenotype [2,3]. *S. pneumoniae* is among the leading causes of community-acquired pneumonia in many countries, affecting mainly children under five years of age and older adults, and remains one of the most important causes of bacterial meningitis. Pneumococcal infections are responsible for more than one million deaths worldwide each year, principally among children and the elderly [4].

The polysaccharide capsule is an essential virulence factor in *S. pneumoniae*; its principal role is to act as a barrier that inhibits phagocytosis by the host’s immune cells [5,6]. The capsule also prevents antibody binding to surface proteins [7], promotes colonization of the nasopharynx [8] and confers resistance to intracellular killing by vascular endothelial cells during invasive disease [9]. The absence of the capsule has been associated with an almost absolute inability of the bacteria to cause invasive infection; therefore, capsular polysaccharides have been used as the basis for pneumococcal vaccines, such as the 23-valent polysaccharide vaccine (PPV23) and conjugate vaccines (PCV), which have demonstrated considerable efficacy in reducing the incidence of pneumonia and invasive disease [10,11]. Although they are immunogenic in immunocompetent individuals, capsular polysaccharides display great structural and serological diversity, with more than 100 distinct serotypes identified so far [12], making fully covered preventive and therapeutic strategies complicated.

Another important virulence factor in pneumococcus is Pneumococcal surface protein A (PspA), a surface-exposed protein noncovalently linked to phosphorylcholine residues in the bacterium’s lipoteichoic and cell wall-associated teichoic acids. PspA displays multifunctional roles, including inhibition of complement activation/deposition on the bacterial surface, interaction with host molecules such as glyceraldehyde-3-phosphate dehydrogenase (GAPDH) and lactate dehydrogenase (LDH), thereby co-opting GAPDH and LDH released by dying host cells for the bacterium’s benefit [13,14], and interference with antimicrobial peptides (AMPs) [15]. Recent studies also suggest that PspA may play a role in membrane remodeling, an essential mechanism for maintaining cellular functionality in response to stress [16]. The importance of PspA is best evidenced by the fact that mutants lacking PspA are highly attenuated in vivo [17,18,19]. Notably, the structure of PspA includes three main regions: a variable N-terminal portion that protrudes from the capsule and is accessible to the host’s immune system, a proline-rich region which connects the N- and C-termini and includes protective epitopes [20], and a more conserved C-terminal region, responsible for anchoring the protein to the bacterial surface. Based on variations in a region within the N-terminal domain called the clade-defining region (CDR), PspA has been classified into three families and six clades [21]. Variation in the host-factor binding domains within the N-terminal also means that PspA has different roles in different strains [13,14].

As PspA is exposed on the bacterial surface, is prevalent among clinical isolates, and plays an important role in pathogenesis, it has been evaluated as a potential serotype-independent vaccine candidate against pneumococcal infections, with encouraging results [22,23]. It is highly immunogenic, induces specific antibodies both in convalescent patients and in experimental models, and protects against pneumonia and invasive disease in different animal models.

Antimicrobial peptides (AMPs) are critical components of the innate response against Gram-positive bacteria, such as pneumococcus. Human defensins, particularly human neutrophil peptides (HNPs), such as HNP-1, are secreted by neutrophils and possess antiviral and antibacterial activities [24]. Research indicates that the interaction between cationic AMPs and the negatively charged bacterial surface results in membrane depolarization and pore formation, leading to membrane disruption [24,25]. In addition to this direct membrane permeabilization, some defensins can also interfere with cell wall synthesis through interaction with Lipid II [26]. Additionally, human α-defensins have been shown to neutralize pneumococcal virulence by inhibiting cholesterol-dependent cytolysins, thereby reducing toxin-mediated cell damage [27]. To counteract these factors, pneumococcus has developed effective strategies to resist AMPs, including the use of efflux pumps, sequestration of antimicrobial molecules, and modifications to the cell wall that alter the capsule structure [28].

While the individual contributions of the polysaccharide capsule and PspA to pneumococcal resistance against some antimicrobial peptides (AMPs) have been established [29,30,31,32], their combined or potentially additive role in protecting against human defensins remains a critical knowledge gap. Notably, the role of the capsule in pneumococcal resistance to HNP-1 has yielded conflicting findings, with some studies reporting a protective effect and others indicating increased susceptibility [33,34], and the specific contribution of PspA on the activity of defensins has yet to be elucidated. Therefore, the present study investigates the individual and combined contributions of PspA and the polysaccharide capsule to pneumococcal resistance against the human neutrophil peptide HNP-1.

## 2. Results

### 2.1. PspA Contributes to Resistance Against HNP-1

**Figure 1:**
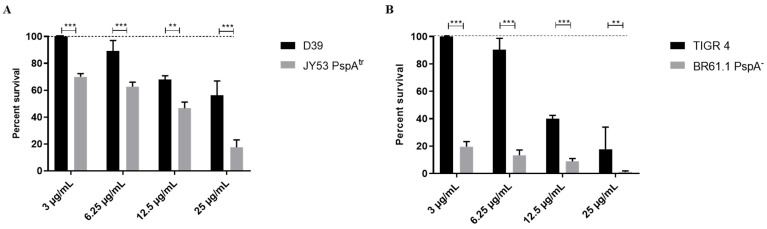
PspA expression affects *Streptococcus pneumoniae* resistance to HNP-1. The bacteria were treated with increasing concentrations of the AMP (3, 6.25, 12.5 and 25 µg/mL) and survival was compared among groups using two-way ANOVA with Sidak post-test. Data represents two independent experiments, each performed with four replicates per group. Each bar represents the percent survival in relation to untreated controls (dashed lines). (**A**) Comparison between wild-type serotype 2 strain (D39) and its isogenic PspA mutant, JY53. (**B**) Comparison between wild-type serotype 4 strain (TIGR4) and its isogenic PspA mutant, BR61.1. ** *p* < 0.01; *** *p* < 0.001 when comparing different bacteria treated with the same concentration of HNP-1.

To assess the role of PspA in resistance to HNP-1, wild-type strains and their corresponding PspA-negative mutants were exposed to different concentrations of HNP-1 for one hour, and bacterial viability was determined by plate count. Figure 1 shows the percent survival of the wild-type strains of serotypes 2 and 4, in comparison with their PspA-negative isogenic mutants, after treatment. In both strains, the absence of PspA resulted in significantly lower resistance to all concentrations of HNP-1, in a dose-dependent manner. For serotype 2 (strain D39 expressing Family 1, Clade 2 PspA) the wild-type strain was highly resistant to peptide concentrations of 3 and 6.25 µg/mL (Figure 1A) with no or marginal bacterial killing, while the PspA-deficient mutant showed significant reductions in viability, with 30 to 40% killing. At 12.5 µg/mL of peptide, 30% killing was observed in the parental strain versus 50% in the mutant strain, and at the highest concentration of HNP-1 less than 50% killing was observed in the parent strain versus over 80% in the PspA-deficient strain.

For serotype 4 (TIGR4 strain expressing Family 2, Clade 4 PspA) and its PspA-negative mutant BR61.1, the effect of HNP-1 was considerably greater at lower concentrations and the bacterium was overall more sensitive to killing. The PspA-negative strain exhibited an over 80% reduction in viability already at the lowest AMP concentration (3 µg/mL). At this concentration no reduction in viability was observed for the parental strain (Figure 1B). Only at 12.5 µg/mL the wild-type parental strain showed significant bacterial killing (approx. 60%) and at this concentration HNP-1, the mutant strain, was almost completely abrogated (Figure 1B). Taken together, these findings indicate that PspA is important for protection against HNP-1, and this varies between strains; however, other factors are also important.

### 2.2. Recombinant PspA Neutralizes the Bactericidal Activity of HNP-1

**Figure 2:**
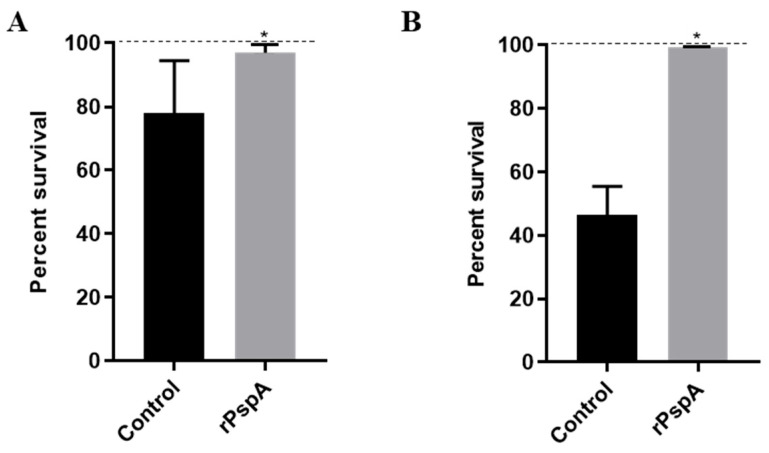
Exogenous PspA limits *Streptococcus pneumoniae* killing by HNP-1. *Streptococcus pneumoniae* D39 (**A**) and its isogenic PspA-negative mutant, JY35 (**B**), were treated with 25 µg/mL of HNP-1 previously incubated with a recombinant N-terminal PspA fragment (rPspA). The control group was incubated with HNP-1 and BSA. Data represents two independent experiments, each performed with four replicates per group. Each bar represents percent survival in comparison with untreated bacteria (dashed line). Statistical analysis was performed using Student *t*-test. * *p* < 0.05 in comparison with the control, incubated with HNP-1 and BSA.

Given the increased susceptibility of PspA-negative strains to HNP-1, we next investigated whether free recombinant PspA (rPspA) could neutralize its observed bactericidal effect. To test this, HNP-1 (25 µg/mL) was pre-incubated with rPspA fragments (from strain P94 expressing the same Family 1, Clade 2 PspA as strain D39) and added to D39 and its isogenic mutant, JY53. As shown in Figure 2A, the addition of rPspA to HNP-1 resulted in a modest but significant increase in D39 survival, compared to bacteria treated with HNP-1 and bovine serum albumin (BSA) as a control protein. A similar effect was observed with the PspA-negative strain (Figure 2B), indicating that free PspA can rescue the bacteria from killing by HNP-1. These results demonstrate the protective effect of bacteria-free PspA, against HNP-1-mediated killing.

### 2.3. Anti-PspA Antibodies Sensitize S. pneumoniae to Killing by HNP-1

**Figure 3:**
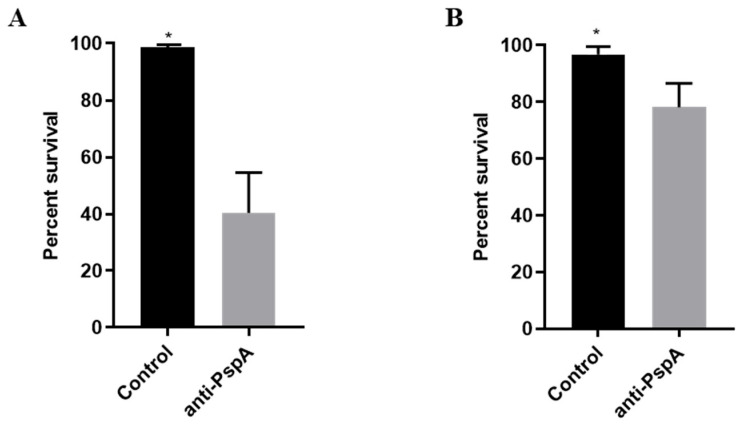
Anti-PspA antibodies enhance *Streptococcus pneumoniae* killing by HNP-1. The peptide was added to *Streptococcus pneumoniae* D39 (**A**) or TIGR4 (**B**) previously opsonized with 10% sera from mice immunized with rPspA, at a concentration of 25 µg/mL. The control group was incubated with sera from mice injected with adjuvant diluted in saline, and HNP-1. Data represents two independent experiments, each performed with four replicates per group. Each bar represents percent survival in comparison with the control. Statistical analysis was performed using Student *t*-test. * *p* < 0.05 in comparison with control serum.

To assess the influence of anti-PspA antibodies on the bactericidal activity of HNP-1, D39 and TIGR4 were opsonized with sera from mice previously immunized with recombinant PspA from strain P94 (Family 1) or PspA P490 (Family 2) prior to peptide exposure. This treatment of D39 with anti-PspA1 resulted in a significantly enhanced bacterial killing compared to bacteria opsonized with control serum and then treated with HNP-1, as shown in Figure 3A. Interestingly, the control serum exerted a protective effect against HNP-1 killing, resulting in increased survival when compared with treatment with HNP-1 alone. Therefore, this serum was used as a control for the experiment. A more modest, but still significant effect was observed in TIGR4 pre-opsonized with anti-PspA2. These results indicate that anti-PspA antibodies favor the bactericidal action of HNP-1 in pneumococci of two different serotypes.

### 2.4. The Role of the Capsule in Pneumococcal Resistance to HNP-1 Is Serotype-Dependent

**Figure 4:**
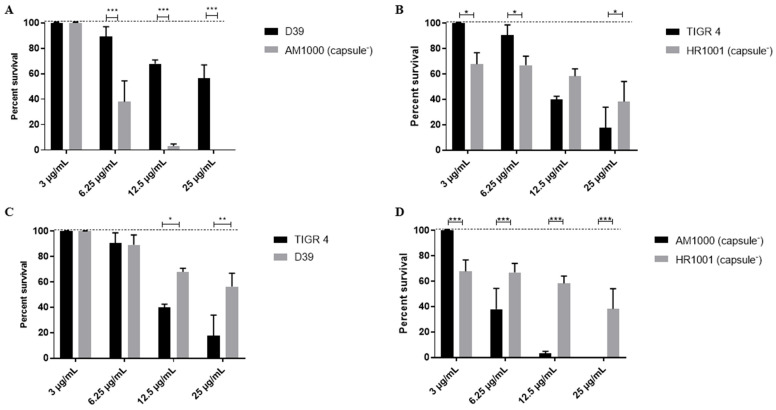
Effects of capsule on *Streptococcus pneumoniae* resistance to HNP-1. The bacteria were treated with increasing concentrations of the AMP (3, 6.25, 12.5 and 25 µg/mL) and survival was compared among groups using two-way ANOVA with the Sidak post-test. Data represents two independent experiments, each performed with four replicates per group. Each bar represents the percent survival in relation to untreated controls (dashed lines). (**A**) Comparison between wild-type serotype 2 strain (D39) and its isogenic capsule negative mutant, AM1000. (**B**) Comparison between wild-type serotype 4 strain (TIGR4) and its isogenic capsule negative mutant, HR1001. (**C**) Comparison between two pneumococcal serotypes, 2 (D39) and 4 (TIGR4). (**D**) Comparison between the two capsule-negative mutants, AM1000 and HR1001. * *p* < 0.05; ** *p* < 0.01; *** *p* < 0.001 when comparing different bacteria treated with the same concentration of HNP-1.

D39 and TIGR4 have distinct capsular types, and we next sought to determine the contribution of the polysaccharide capsule in HNP-1 resistance. This was done by treating wild-type strains and their isogenic capsule-negative mutants with increasing concentrations of the AMP. For the serotype 2 strain a protective role of capsule was observed when comparing the wild-type D39 strain to its non-encapsulated mutant, AM1000 (Figure 4A). The wild-type strain was significantly more resistant to HNP-1 treatment than the capsule-negative mutant at all concentrations above 3 µg/mL. At a concentration of 6.25 µg/mL, the capsule-negative strains showed a viability reduction of 40% (compared to 10% for the wild-type strain), with 100% bacterial killing measured at the highest concentrations (12.5 and 25 µg/mL) when the wild-type was reduced by 35% and 40%, respectively.

On the other hand, the effects of HNP-1 treatment in the serotype 4 strain (TIGR4) and its isogenic capsule-deficient mutant (HR1001) varied depending on the peptide concentration. The parental strain was significantly more resistant than the mutant in the lower concentrations of HNP-1 (3 and 6.25 µg/mL), while it tended to become more susceptible than the mutant as the HNP-1 concentration increased. At the highest concentration of the peptide (25 µg/mL), the mutant strain was significantly more resistant to the AMP than the parent strain (Figure 4B).

A comparison between the wild-type strains, D39 (serotype 2) and TIGR4 (serotype 4), revealed marked differences in susceptibility to the peptide (Figure 4C). Although both strains were resistant to the lower concentrations of HNP-1, the D39 strain was significantly more resistant than TIGR4 at AMP concentrations of 12.5 and 25 µg/mL. The capsule mutant strains also differed in their susceptibility to killing by HNP-1 (Figure 4D). The capsule-4 derivative, HR1001, was significantly more resistant to AMP concentrations of 6.25 µg/mL and higher than AM1000.

Comparing these results to the PspA-negative data suggests that in strain D39, the capsule provides more resistance to HNP-1 than PspA, whereas in strain TIGR4, the reverse is apparent. This result suggests that the effects of the capsule on pneumococcal resistance to HNP-1 vary among different genetic backgrounds, and this is likely due in part to differences in capsule type.

### 2.5. Free Capsular Polysaccharides Partially Neutralize the Bactericidal Activity of HNP-1

**Figure 5:**
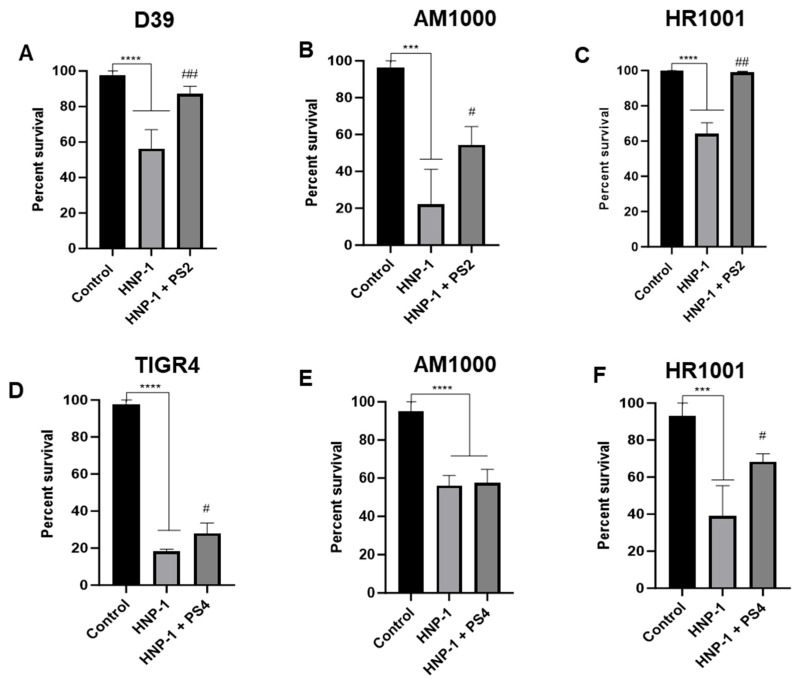
Effects of free capsular polysaccharide in *Streptococcus pneumoniae* resistance to HNP-1. Wild-type strains D39 and TIGR4, and its isogenic capsular-negative mutants AM1000 and HR1001 were treated with 25 µg/mL of HNP-1 alone or in combination with 10 µg/mL of purified PS-2 (**A**–**C**) or PS-4 (**D**–**F**). Data represents two independent experiments, each performed with four replicates per group. Each bar represents the percentage of bacteria surviving treatment, in comparison with the untreated control. Comparison among groups was performed using one-way ANOVA with the Tukey post-test. *** *p* < 0.001 in comparison with untreated control. **** *p* < 0.0001 in comparison with untreated control. ## = *p* < 0.01 in comparison with untreated control. # = *p* < 0.05 when comparing treatment with HNP-1 alone and in presence of purified PS-2 or PS-4.

To determine whether free capsular polysaccharide could neutralize the bactericidal action of HNP-1, the peptide was pre-incubated with either serotype 2 (PS-2) or serotype 4 (PS-4) polysaccharide prior to bacterial exposure. As shown in Figure 5A, HNP-1 pre-treated with free PS led to a significant decrease in D39 killing when compared to treatment with HNP-1 alone: the group incubated with PS-2 showed only 10% killing, compared to over 40% killing after treatment with the peptide alone. A more moderate effect was observed for PS-4 plus HNP-1 treatment in TIGR4 (Figure 5D): HNP-1 alone markedly reduced bacterial viability with over 80%, while HNP-1 combined with PS-4 showed a slight rescue with only 75% killing.

The effect of free polysaccharide was also investigated in capsule-negative mutants AM1000 and HR1001. Addition of free PS-2 significantly protected both capsule-negative mutants from HNP-1-induced killing (Figure 5B,C). On the other hand, the presence of PS-4 rescued only the HR1001 mutant; the AM1000 strain showed similar killing with or without PS-4. Taken together, these results demonstrate that the effect of free-polysaccharides in pneumococcal resistance to HNP-1 are dependent on their biochemical constitution, with PS-2 being more effective in neutralizing the peptide than PS-4.

### 2.6. PspA and Capsule Additively Contribute to HNP-1 Resistance

**Figure 6:**
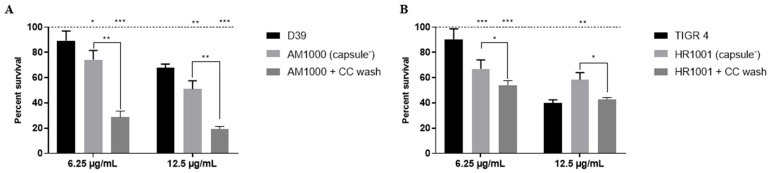
Combined effects of PspA and capsule on *Streptococcus pneumoniae* resistance to HNP-1. Two capsule-negative mutants, AM1000 (**A**) and HR1001 (**B**), were washed with choline chloride to remove PspA and treated with two concentrations of the AMP (6.25 and 12.5 µg/mL), and survival was compared among capsule-producing, capsule-negative, and capsule-negative + PspA removed groups. Data represents two independent experiments, each performed with four replicates per group. Statistical analysis was performed using two-way ANOVA with the Sidak post-test. Each bar represents the percent survival in relation to untreated controls (dashed lines). * *p* < 0.05 in comparison with the parental strain; ** *p* < 0.01 in comparison with the parental strain; *** *p* < 0.001 when comparing intact and cc-washed bacteria.

Finally, and to further investigate the combined effects of PspA and the capsule in pneumococcal resistance to HNP-1, the capsule-negative mutants were washed with choline chloride to remove surface-bound PspA and then exposed to HNP-1. In the serotype 2-derived mutant, AM1000, removal of PspA led to a marked reduction in bacterial viability (Figure 6A), demonstrating a clear additive protective effect between PspA and the capsule for this strain.

In contrast, a more complex interaction was observed in the serotype 4-derived capsule negative strain, HR1001. Removal of PspA rendered the bacterium more susceptible to the lower concentration of the AMP, 6.25 µg/mL. However, when the AMP concentration was enhanced to 12.5 µg/mL, PspA removal did not aid in bacterial killing, presenting similar survival as the wild-type strain, TIGR4 (Figure 6B). Interestingly, there is a significant reduction in bacterial viability when comparing the capsule-negative derivative of TIGR4, HR1001, before and after PspA removal, indicating that the loss of PspA favors HNP-1 activity in this resistant non encapsulated mutant. Taken together, these results reveal that while PspA consistently confers protection, its combined effect with the polysaccharide capsule on HNP-1 resistance is dependent on the pneumococcal serotype.

### 2.7. PspA Shows Potential Electrostatic Interaction with HNP-1 In Silico

**Figure 7:**
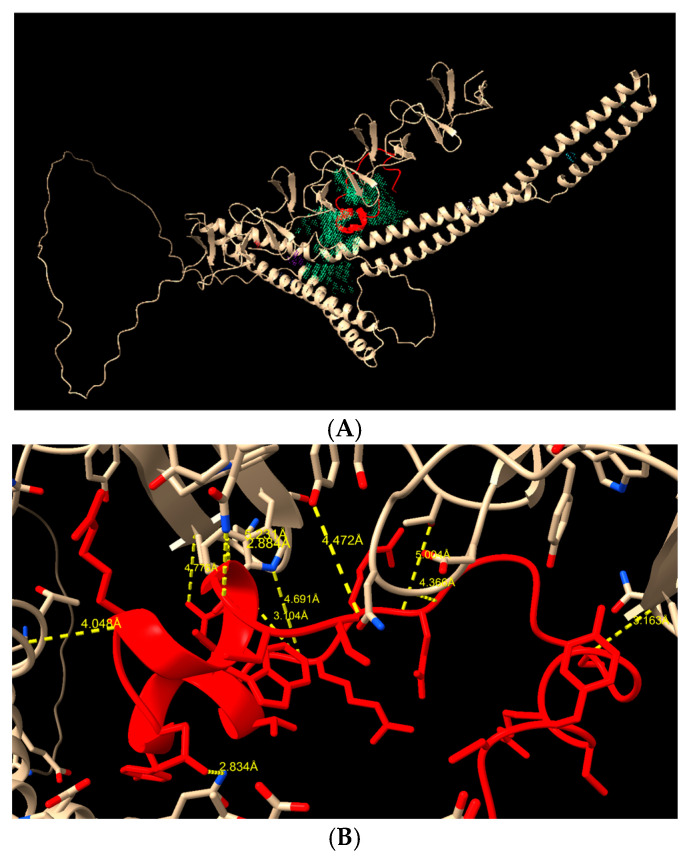
Molecular docking of D39 protein (off-white) and HNP-1 peptide (red). (**A**) Entire protein to show the main cavities and the peptide positioning. (**B**) H-bonds between D39 and HNP-1 (in yellow, showing the binding and atoms distance).

Molecular docking analysis showed that the peptide HNP-1 could bind to all studied isoforms of PspA. Figure 7A shows the D39 structure and the HNP-1 peptide (in red) merged with the main protein cavity (in green), indicating a potential site for interaction and biological activity based on structural and electrostatic complementarity. In Figure 7B, it is possible to see the H-bonds with the protein and the peptide, with three interactions below 3 Å (Gln183–Gln22, Gln477–Arg15, Asn499–Phe28), with low binding energy, calculated to be −6.87 (PepProScore).

**Figure 8:**
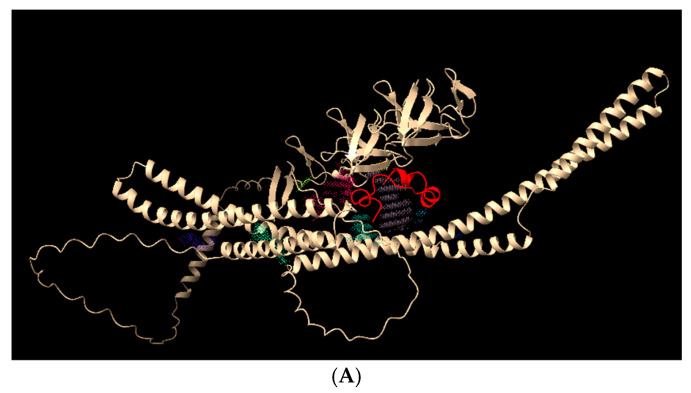

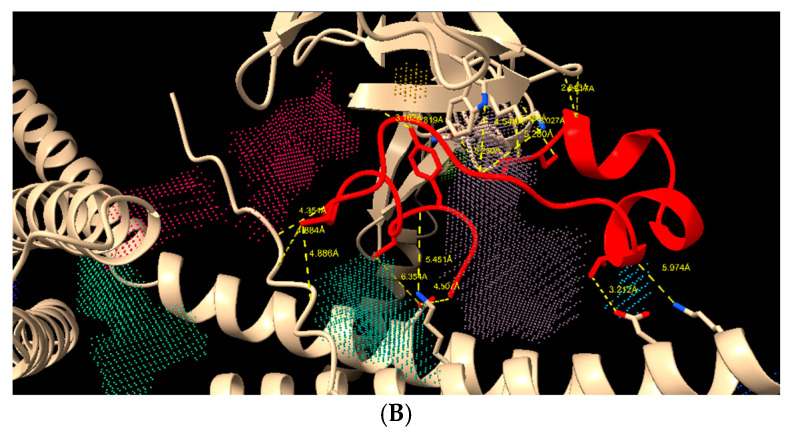
Molecular docking of D39-2 protein (off-white) and HNP-1 peptide (red). (**A**) Entire protein to show the main cavities and the peptide positioning (purple and green shadows). (**B**) H-bonds between D39-2 and HNP-1.

The same pattern could be observed in D39-2: the HNP-1 peptide (in red) bound to D39-2 protein with low energy (−6.36 PepProScore), in the main cavity (purple) (Figure 8A). Four H-bonds ~3 Å were identified, suggesting the plausibility of an association (Figure 8B—Asn468-Ala27, Thr462-Tyr3, Trp464-Tyr3, Asp68-Thr18).

**Figure 9:**
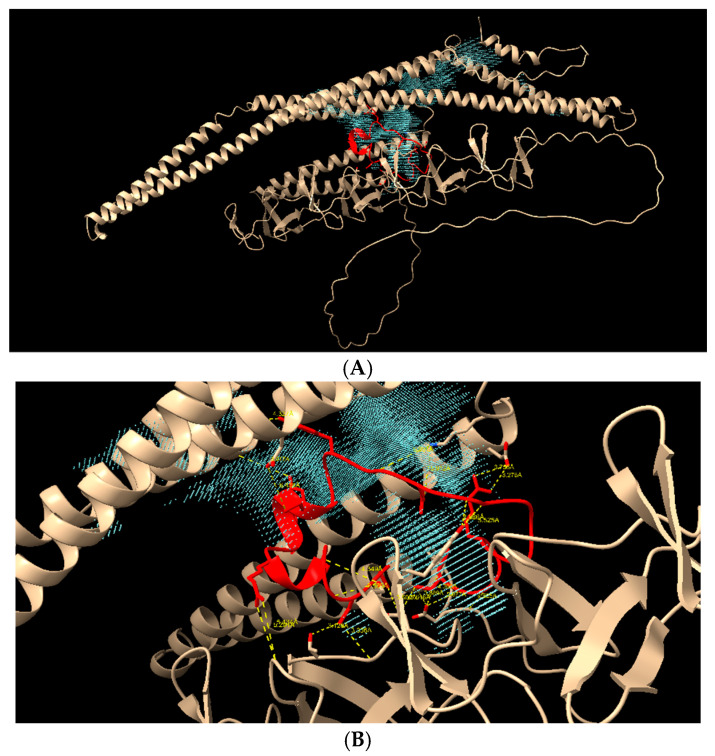
Molecular docking of TIGR4 protein (off-white) and HNP-1 peptide (red). (**A**) Entire protein to show the main cavities and the peptide positioning. (**B**) H-bonds between TIGR4 and HNP-1.

When TIGR4 was analyzed, the peptide was positioned deeper in the pocket, shown in the main cavity of Figure 9A (in blue). Moreover, 21 H-bonds were identified, with 3 below 3 Å (Tyr612-Ala11, Ser615-Phe28 and Thr647-Cys29) (Figure 9B). The energy binding was calculated to be −6.41 PepProScore.

**Figure 10:**
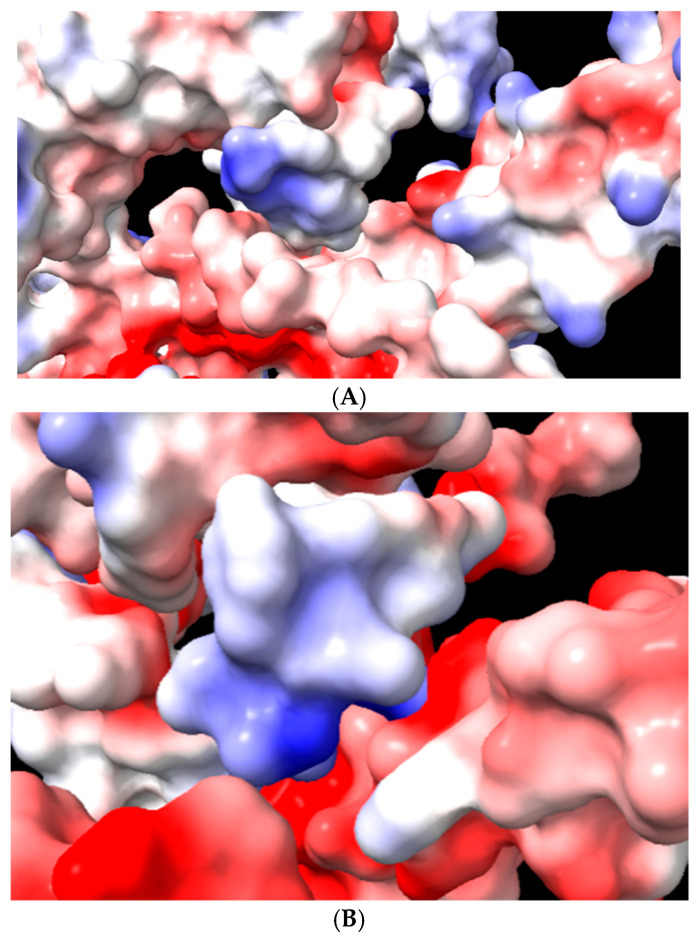
Electrostatic surface potential of PspA isoforms from strain D39 and TIGR4. The molecular surfaces of PspA from strain D39 (**A**) and TIGR4 (**B**) are colored according to their electrostatic potential (red, negative; blue, positive).

The electrostatic potential was mapped onto the molecular surface of protein and the docked peptide to visualize the interaction according to charges (Figure 10). The electrostatic surface potential analysis of PspA isoforms revealed a predominance of acidic regions, indicated by the red-colored negative pockets on both D39 and TIGR4 structures. Since HNP-1 is a highly cationic peptide rich in positively charged residues, these results suggest an electrostatic complementarity that may favor binding of the defensin to the protein surface.

## 3. Discussion

The polysaccharide capsule and the surface protein PspA are recognized as central virulence factors in *Streptococcus pneumoniae*, acting independently and complementarily in immune evasion and resistance to antimicrobial molecules [6,15,35]. Thus, the present study aimed to investigate the individual and combined contributions of PspA and the capsule to the bactericidal action of the human defensin HNP-1.

A comparative analysis of wild-type and PspA-negative mutant pneumococcal strains revealed an increased sensitivity of the mutants to treatment with HNP-1. This effect was independent of capsular type, as shown by the significantly reduced survival of PspA-negative mutants derived from both serotype 2 and serotype 4 strains. These results suggest that PspA plays an important role in protection against the antimicrobial peptide HNP-1, as its absence renders pneumococci more susceptible even in the presence of an intact capsule. Supporting these findings, we demonstrate that addition of a recombinant PspA fragment can rescue pneumococci from the bactericidal activity of the peptide, increasing survival upon HNP-1 treatment. This piece of evidence further reinforces PspA’s protective efficacy, while indicating that the protein does not need to be attached to the bacteria to exert its effect.

Previous work evaluating the interactions of pneumococci with antimicrobial peptides have found that PspA can protect the bacterium from apolactoferrin—the bactericidal form of the defense protein lactoferrin [29,32,36] and the bovine cathelicidin, indolicidin [30]. In both cases, PspA has been shown to specifically bind to the antimicrobial proteins [30,37]. These studies demonstrate that PspA exhibits broad-spectrum AMP-sequestering activity, which, together with its well-documented ability to inhibit complement activation, contributes to pneumococcal survival during both colonization and invasive disease.

Since PspA has long been investigated as a potential vaccine candidate against pneumococcal disease, we sought to determine how anti-PspA antibodies affect HNP-1-mediated killing. Pre-opsonization with serum from mice which had been immunized with recombinant N-terminal PspA fragments enhanced the bactericidal effect of HNP-1 in comparison with control serum, likely by inhibiting PspA’s capacity to bind the peptide, thereby allowing HNP-1 to access the bacterial membrane and exert its disruptive effect. These observations support the notion that antibodies induced by vaccination with recombinant N-terminal fragments of PspA may confer protection not only by promoting opsonophagocytosis [38], but also by unmasking the action of innate immune effectors such as antimicrobial peptides, thereby enhancing bacterial clearance at mucosal and systemic sites. Furthermore, the effect of anti-PspA serum in promoting HNP-1 killing was demonstrated with pneumococci of two different genetic backgrounds and serotypes, suggesting that it is not strain-dependent.

Regarding the capsule, we observed that serotype 2 (D39) showed greater resistance to HNP-1 than serotype 4 (TIGR4). Additionally, the loss of the capsule had variable effects depending on the bacterial serotype: the capsule negative mutant derived from serotype 2 showed reduced survival in comparison with the parental strain, while conversely, the serotype 4 parental strain (TIGR4) was more resistant at lower AMP concentrations but became significantly more susceptible that its non-encapsulated mutant at the highest concentration. These results indicate that it is not merely the presence of the capsule, but its composition that has a profound impact on the pneumococcal ability to resist the bactericidal action of the human defensin HNP-1.

Importantly, the conclusions drawn in the present study are restricted to the genetic backgrounds analyzed, namely strains D39 (serotype 2) and TIGR4 (serotype 4), which are widely used experimental models but do not encompass the full diversity of pneumococcal capsule structures. The atypical phenotype observed for the TIGR4 capsule mutant at higher HNP-1 concentrations highlights that capsule-mediated effects may vary according to strain-specific factors beyond serotype alone. Therefore, our findings should be interpreted as a proof-of-concept demonstrating that capsule composition and PspA expression can differentially modulate HNP-1 susceptibility, rather than a generalizable feature applicable to all pneumococcal serotypes. While systemic HNP-1 levels are in the ng/mL range, local concentrations at infection sites are significantly higher. In adults, defensin levels at epithelial interfaces reach 10–100 µg/mL, while concentrations within neutrophil granules can exceed 10 mg/mL. Accordingly, the HNP-1 range used in this study accurately reflects the physiological pressure encountered by *S. pneumoniae* during active infection [39].

Insights into the possible mechanisms underlying this effect suggest that the surface electric charge may help shield the bacterium from cationic peptides such as HNP-1. Previous data by our research group have shown that denser capsules with a higher negative charge offer greater protection against indolicidin, a cationic peptide belonging to the cathelicidin family [31]. Accordingly, the serotype 2 strain D39 has a much higher electronegativity than the capsule 4 strain, suggesting that the capsule charge may influence pneumococcal resistance to different cationic antimicrobial peptides. A similar protective effect of the polysaccharide capsule against AMPs has been demonstrated by Llobet et al. [33]. In that study, the addition of negatively charged pneumococcal polysaccharide, but not neutral or positive molecules, protected unencapsulated bacteria from killing by HNP-1. Moreover, addition of polycations restored AMP activity, indicating that the negative charge is essential for the protective effects of polysaccharides. The authors propose that the capsule may act as a “decoy” by attracting the peptides away from the membrane, thus preventing their bactericidal activity. Our findings, therefore, suggest that the effect of the capsule on the action of HNP-1 is dependent on both the serotype and the concentration of the peptide, which is in line with recent studies that highlight the structural diversity of capsular polysaccharides and their impact on colonization and immune response [40,41,42].

Contrasting those results, Beiter et al. (2008) [34] observed that non-encapsulated mutant pneumococci were more resistant to treatment with human defensins than their respective parental strains. This variation could be attributed to differences in the peptide: the authors used a mixture of HNP-1 to III purified from human neutrophils, instead of isolated HNP-1. It is also important to notice that in that study, the D39 strain did not show reduced resistance to the AMP, but rather a comparable survival to the capsule negative derivative.

Another relevant point was the observation that purified polysaccharides (PS-2 and PS-4) were able to partially neutralize the activity of HNP-1. This effect was more evident with PS-2, suggesting a serotype-specific role in modulating resistance. This mechanism is compatible with the molecular “decoy” hypothesis described by Llobet et al. (2008), in which bacterial capsules, even in free form, act by diverting antimicrobial peptides and reducing their efficacy [33]. That study has also shown that AMP treatment promotes capsule release, resulting in an entrapment of the peptide to prevent killing. Further studies have confirmed that capsule shedding promoted by cationic AMPs in the mucosal sites is an important mechanism for pneumococcal colonization, allowing the exposure of adhesins at the bacterial surface that favor interaction with epithelial cells, while the free polysaccharides sequester the AMPs and prevent killing [43]. The mechanism of capsule shedding is dependent on the cell wall hydrolytic activity of the suicidal amidase autolysin LytA, which promotes a restructuring of the bacterial surface and favors epithelial invasion [43].

To evaluate the additive contributions of capsule and PspA to pneumococcal resistance against HNP-1, two capsule-negative mutant strains were incubated with choline chloride to remove surface-attached PspA, prior to treatment with the peptide. The combined analysis revealed that the capsule and PspA both play a role in protecting against HNP-1, especially in serotype 2, where the absence of the capsule and PspA greatly impaired survival. In serotype 4, although the capsule mutant strain showed increased resistance to the higher concentration of HNP-1, PspA removal still significantly reduced survival, suggesting that resistance to HNP-1 results from a dynamic equilibrium between multiple surface factors. This variation may be associated with both biochemical differences of capsular polysaccharides and differential expression of cell wall-associated proteins, as described by Mitchell (2010) and Ganaie et al. (2025) [5,12]. Although treatment with choline chloride affects other choline-binding proteins like PspC and CbpA, PspA remains the most abundant choline-binding protein in pneumococci. Furthermore, the reduction in survival observed following chemical wash closely mirrors the susceptibility profile displayed by PspA–isogenic mutants when exposed to HNP-1, while the addition of soluble rPspA fragments was able to neutralize the peptide’s action and independently rescue bacterial viability, demonstrating that PspA confers protection regardless of other CBPs removed by the treatment. This specificity is further corroborated by previous studies involving other cationic antimicrobial peptides with similar properties, such as apolactoferrin and indolicidin, where it was established that the absence of other abundant CBPs, like PspC and PcpA, does not significantly alter the bacterium’s resistance to death by these peptides [30,32].

Although our study functionally demonstrates the contribution of both capsule and PspA to HNP-1 resistance, the underlying molecular mechanisms remain speculative. Potential mechanisms that could prevent the peptide from reaching the bacterial membrane include the direct sequestration of HNP-1 by surface components, altered peptide aggregation, charge shielding or reduced effective concentration. Molecular docking analysis provides hypothesis-generating insights into the predicted interactions between PspA and HNP-1 in silico. The electrostatic surface potential analysis of PspA (strains D39 and TIGR4) reveals the presence of distinct negatively charged pockets that likely exert a strong electrostatic attraction on the highly cationic HNP-1 peptide. This surface mapping provides a biophysical rationale for the predicted associations observed in our molecular docking analysis, indicating that the protein surface offers a favorable environment for peptide entrapment. Rather than confirming a specific biochemical bond, these results suggest a charge-based mechanism for peptide interactions, consistent with the ‘electrostatic decoy’ model proposed for other pneumococcal surface factors.

This computational observation supports the functional survival data observed in the bactericidal assays. However, the present data are not sufficient to determine the exact mechanism of interaction, and future studies employing different techniques are necessary to clarify the physical basis of these potential associations and provide a detailed mechanism basis for the observed resistance. Recognition of these mechanisms is essential for understanding the pathogenesis of pneumococcus and for developing alternative therapeutic strategies. In this sense, the combination of defensins with agents that inhibit capsular synthesis or block the action of PspA may represent a promising approach in the face of increasing antimicrobial resistance [44,45]. Also, while our findings provide consistent trends, the limited number of biological replicates is a constraint that may affect the statistical power of multi-factor comparisons.

In conclusion, our results reinforce the multifaceted role of capsule and PspA in the resistance of *S. pneumoniae* against human defensins and indicate that future prevention and treatment strategies should consider not only serotype variability, but also the contribution of surface proteins in the modulation of host response.

## 4. Materials and Methods

### 4.1. Bacterial Strains and Growth Conditions

Table 1 presents detailed information on the pneumococcal strains and mutants used in this study. The bacteria were kept as frozen stocks at −80° and, on the day preceding the experiments, plated on blood agar (BA; Laborclin, Pinhais, Brazil) and incubated for 18–20 h at 37 °C in microaerophilic conditions. The next day, bacterial colonies were transferred to 5 mL of Todd Hewett broth (Conda Laboratories, Madrid, Spain) supplemented with 0.5% yeast extract (THY; Sparks, MD, USA) and cultured until they reached an optical density (O.D._600nm_) of 0.3–0.4 (approximately 10^7^ CFU/mL). The mutant strains are part of a collection maintained at the University of Alabama at Birmingham (UAB) and kindly provided by Dr Anders Hakansson from Lund University (LU).

### 4.2. In Vitro Bactericidal Assay

Pneumococcal susceptibility to HNP-1 (ANASPEC code AS-60743, Fremont, CA, USA) was determined using the in vitro bactericidal assay adapted by Waz et al. 2024 [30]. Once the cultures reached the desired O.D._600nm_, 2 mL of the culture was pelleted by centrifugation at 5000 rpm for 5 min, washed with 1 mL of PBS and resuspended with an equal volume of sterile 10 mM sodium phosphate buffer at pH 7.6 [51,52]. The bacterial suspension was then subjected to treatment with different concentrations (ranging from 3 to 25 µg/mL) of HNP-1, and dissolved in 10 mM sodium phosphate buffer (pH 7.6) and kept at −20 °C. The untreated group was incubated with phosphate buffer alone. After one hour incubation at 37 °C, the samples were subjected to serial dilution, plated on BA and incubated at 37 °C for 16–20 h. The number of surviving colony-forming units of bacteria after treatment was calculated and compared among the groups.

To assess the contribution of the polysaccharide capsule to HNP-1 bactericidal activity, wild-type pneumococci and isogenic mutants that do not produce capsules were treated with increasing concentrations of the peptide and plated as described. Similarly, the effect of PspA expression on pneumococcal susceptibility to HNP-1 was evaluated by comparing wild-type strains D39 and TIGR4 with their isogenic Δ*pspA* mutants (JY53 and BR61.1, respectively).

### 4.3. Recombinant PspAs

The PspA fragment used in this study was derived from strain P94 (Family 1, Clade 2) [38]. The gene fragments encompassing the sequences for the N-terminal alpha-helical region and the proline-rich domain (Supplementary Figure S1) were amplified from the chromosomal DNA and inserted into the pAE-6xHis vector. Protein expression was carried out in *E. coli* BL21 (DE3), followed by purification via affinity chromatography, following the method outlined by Goulart et al., 2017 [38]. Contaminating LPS was removed from the purified proteins using the protocol described in Liu et al. [53].

### 4.4. Production of Anti-PspA Antibodies

Female BALB/c mice (6 weeks) were immunized via subcutaneous injection with 10 μg of rPspA in three consecutive doses at 10-day intervals, using Alum (Al(OH)_3_, 50 μg/animal/dose) as an adjuvant. Control mice were injected with the adjuvant diluted in saline solution. After the final immunization, 100 μL of blood was collected through retro-orbital puncture after local anesthesia. Pooled sera from five mice immunized with the same protein were separated by centrifugation and stored at −20 °C for subsequent analysis. Specific IgG levels were determined by ELISA.

### 4.5. Effect of rPspA and Purified PS Addition on HNP-1 Activity

The effect of soluble PspA and capsular polysaccharide on pneumococcal susceptibility to HNP-1 was evaluated by adding 10 µg of rPspA or purified PS to the AMP solution prior to incubation with the bacteria. The control group was incubated with 10 µg of bovine serum albumin (BSA; Sigma-Aldrich, St. Louis, MO, USA) and 25 µg/mL of HNP-1. The number of surviving bacteria was measured in each group after plating on BA and incubation for 16 h at 37 °C.

### 4.6. Effect of Anti-PspA on Pneumococcal Killing by HNP-1

To determine the effect of anti-PspA antibodies on pneumococcal killing by HNP-1, the bacteria were opsonized with 10% of anti-rPspA serum (1 mg/mL) for 15 min at 37 °C prior to treatment with 25 µg/mL of HNP-1 (the serum concentration was based on previous studies performed by the group [30]). The control group was pre-incubated with serum from sham-immunized mice before treatment. The number of surviving bacteria was measured in each group after plating and incubation for 16 h.

### 4.7. Choline Chloride Treatment to Remove Surface Proteins

PspA is anchored to the bacterial cell wall via choline residues, thus being classified as a choline-binding protein. Chemical removal of choline binding proteins is achieved by washing the bacterial pellets with a 2% choline chloride solution (Sigma-Aldrich, St. Louis, MO, USA) [30]. To evaluate the combined effects of the polysaccharide capsule and PspA on the action of HNP-1, the capsule-negative mutant strains were grown as described and harvested by centrifugation and divided into two groups. The treatment group was resuspended in 2% choline chloride diluted in PBS and incubated for 10 min at room temperature, while the control group was incubated in PBS alone for the same duration. Following incubation, cells were washed twice with PBS to remove residual choline chloride and the eluted proteins, before being immediately used in the bactericidal assay. This protocol has been shown to effectively release PspA from the bacterial surface [37], as confirmed by Western blot analysis of cc-wash [32].

### 4.8. Molecular Docking

PspA proteins (D39 or TIGR4) were modeled using the Swiss Model workspace (Computational Structural Biology Group at the SIB Swiss Institute of Bioinformatics and the Biozentrum of the University of Basel) [54] from the amino acid sequence available in Uniprot. These models (.pdb) were inserted in MDockPep with the HNP-1 FASTA sequence. The first model, with the lowest binding energy, was selected to be analyzed using UCSF ChimeraX 1.10 [55] to determine the cavities of the protein and the positioning of the peptide, as well as H-bonds between the protein and the peptide, to verify amino acid binding and its distance. Moreover, using ChimeraX 1.10, the electrostatic potential of both the protein and peptide was calculated after the docking results, which were determined by default charges and atom types from the Assisted Model Building with Energy Refinement (AMBER) force field, based on residues Coulombic values. 

### 4.9. Statistical Analysis

All experiments were performed with four independent samples per treatment group (*n* = 4) and were independently replicated once or twice to ensure data reproducibility. The comparison between wild-type and mutant pneumococcal strains treated with different concentrations of the AMP was performed using two-way ANOVA with Sidak’s post-test. Differences in survival between bacteria treated with recombinant protein and control protein or with control serum and anti-PspA serum were analyzed using Student’s *t*-test. Statistical significance was calculated based on *p* ≤ 0.05. All graphs were generated using GraphPad Prism 8.

## Figures and Tables

**Table 1:** Pneumococcal strains used in this study. UAB—University of Alabama at Birmingham, USA.

Strain	Serotype	PspA Clade	Strain Mutation	Source	Reference
D39	2	2	Wild-type	UAB	[46]
JY53	2	-	pKSD300 × D39; Emr (PspA^tr^)	UAB	[47]
AM1000	_	2	D39 DcpsABSCETFGHI (Capsule^−^)	UAB	[48]
TIGR4	4	4	Wild-type	UAB	[49]
BR61.1	4	_	pREN3 × TIGR4 (PspA^−^)	UAB	[50]
HR1001	_	4	TIGR4 DcpsABSCETFGHI (Capsule^−^)	UAB	[48]

## Data Availability

The original contributions presented in this study are included in the article/Supplementary Material. Further inquiries can be directed to the corresponding author.

## Supplementary Materials

The following supporting information can be downloaded at: https://www.mdpi.com/article/10.3390/ijms27072975/s1.
