# Supplementary figures and images for: Capsule and PspA Cooperatively Confer Resistance of Streptococcus pneumoniae to the Human Defensin HNP-1
Source: Int J Mol Sci. 2026 Mar 25;27(7):2975. doi: 10.3390/ijms27072975 (PMC13073006; doi:10.3390/ijms27072975)

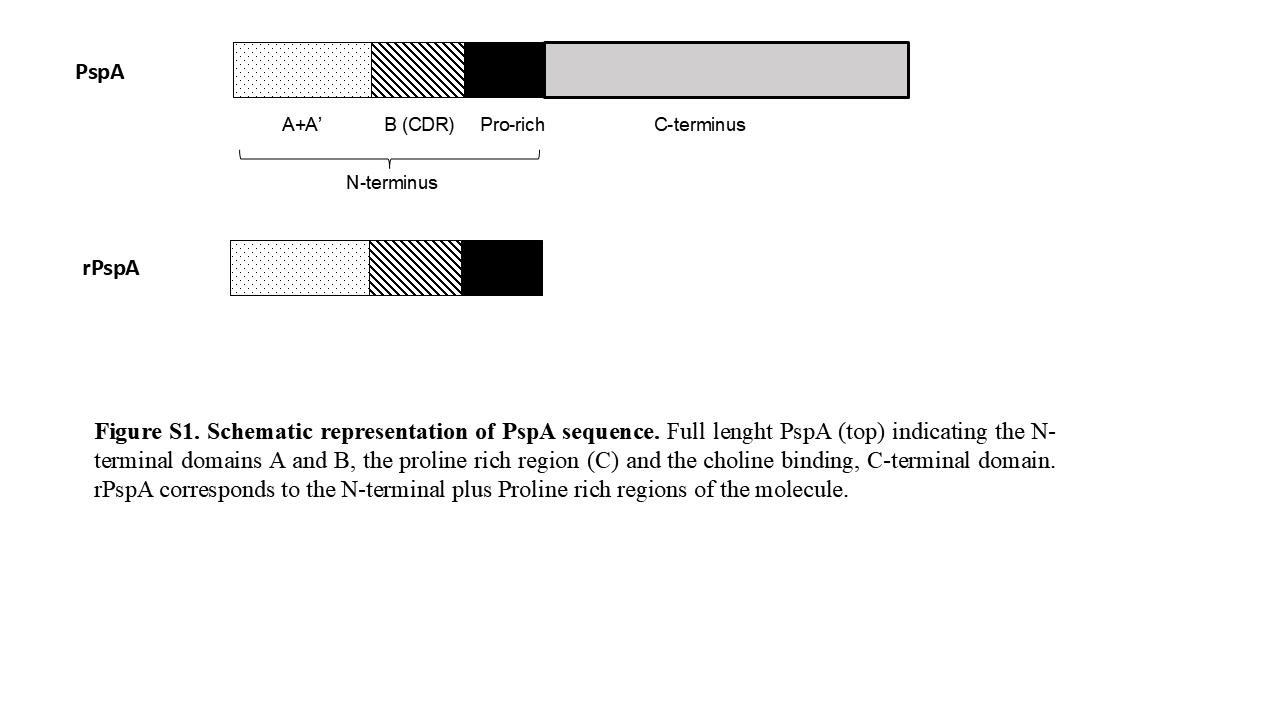

Supplement: Supplementary file 1 [file ijms-27-02975-s001.zip › ijms-3976989-supplementary.tif]
